# Development of an Efficient Recombinant Mosquito Densovirus-Mediated RNA Interference System and Its Preliminary Application in Mosquito Control

**DOI:** 10.1371/journal.pone.0021329

**Published:** 2011-06-16

**Authors:** Jinbao Gu, Min Liu, Yuhua Deng, Hongjuan Peng, Xiaoguang Chen

**Affiliations:** Key Laboratory of Prevention and Control of Emerging Infectious Diseases of Guangdong Higher Education Institutes, School of Public Health and Tropical Medicine, Southern Medical University, Guangzhou, Guangdong, China; University of Kansas, United States of America

## Abstract

The *Aedes aegypti* densovirus (AeDNV) has potential as a delivery vector for foreign nucleic acids into mosquito cells. In this study, we investigated the ability of plasmids containing recombinant viral transducing genome to induce RNA interference (RNAi) effects in C6/C36 cells. We then evaluated the efficiency of a recombinant AeDNV vector to induce RNAi in *Aedes albopictus* larvae. We found that the expression of *V-ATPase* was inhibited by up to 90% at 96 h post-transfection in transfected C6/C36 cells. In addition, the bioinsecticidal activities of various RNAi-expressing AeDNV vectors used to infect *Ae. albopictus* larvae were also tested. We found that when *Ae. albopictus* larvae were infected with recombinant AeDNV, expression of *V-ATPase* was downregulated by nearly 70% compared to controls. Furthermore, the median survival time bioassays demonstrated that recombinant AeDNV caused more serious pathogenic effects than the wild type virus. This is the first report showing that recombinant virus plasmid and corresponding recombinant AeDNV can be used as an effective in vitro and in vivo RNAi delivery system, respectively.

## Introduction

Mosquito-borne diseases are a major international public health problem that continue to pose a public health threat [1]. Chemical insecticides, which have traditionally been used in response to epidemics, are a major part of sustainable, integrated mosquito management for the prevention of mosquito-borne diseases. However, such strategies have proven to be relatively ineffective or undesirable as a result of to the development of resistance within mosquito populations and the negative environmental impacts [2], [3]. In light of these problems, the search for new alternative approaches that could be applied to combat the spread of these diseases continues.

The technique of RNA interference (RNAi) is a powerful means of suppressing the expression of specific genes and, as such, provides a powerful new tool for the investigation of gene function [4]. In fact, RNAi offers a great deal of potential for successful mitigation of various crop pest insects [5]–[7]. Although the dsRNA-mediated silencing of essential genes in insects can induce antifeedant effects and, ultimately, morbidity, the efficient uptake of dsRNA by oral or topical applications is required [5]. Unfortunately, in the case of mosquitoes, *in vivo* RNAi delivery has thus far relied on microinjection [8]. Microinjection is highly technically demanding and time-consuming, and is therefore not suitable for high-throughput genetic analyses or practical applications, including mosquito control.

Mosquito densoviruses (MDV; family *Parvoviridae*, genus *Brevidensovirus*) are non-enveloped, single-stranded DNA viruses, which are relatively stable in the environment and have the potential to spread and persist naturally in mosquito populations. MDVs can cause systemic infection in mosquitoes and replicate in many different tissues, including the midgut, malpighian tubules, fatbody, musculature, neurons, and salivary glands [9]. Plasmid-based, infectious clones of MDV can be constructed by inserting the intact genome into a plasmid. Once these clones are transfected into mosquito cells, the viral genome can be released from the plasmid vector and infectious viral particles are produced. Using plasmid vectors, the viral genome can easily be manipulated and recombinant viruses can be generated *in vitro* by transfecting cultured mosquito cells with helper virus [10], [11]. These characteristics make MDV a valuable transducing agent in mosquito biology.

In the present study, we developed a recombinant *Aedes aegypti* Densovirus (AeDNV) siRNA expression system that utilized artificial introns and a putative mosquito U6 snRNA promoter-driven siRNA expression cassette. The endogenous *V-ATPase* gene of the Asian dengue fever mosquito, *Ae. Albopictus*, was targeted for silencing both *in vitro* and *in vivo*, and bioassays were carried out *in vivo* to measure the effect of recombinant viruses on larval survival.

## Materials and Methods

### siRNAs

Five candidate siRNAs against the gene encoding the *Ae. albopictus V-ATPase* subunit A were generated using siRNA design tools (Dharmacon, USA) and analyzed by a BLAST search to ensure that they did not have significant sequence homology with other genes. The most effective two siRNAs, as confirmed by the RT-PCR analysis of transfected C6/36 cells, were selected to construct the siRNA expression vector. The two siRNAs (siRNA1 and siRNA2) corresponded to the coding regions at 1,418–1,436 and 309–327 nucleotides, respectively, of the *V-ATPase* subunit A mRNA (Accession no. AY864912). The control RNA (scRNA, CGACGACTATCGTGCAATT) consisted of a unique sequence that did not match any sequence in the genome of interest.

### Plasmid construction

**Figure 1:**
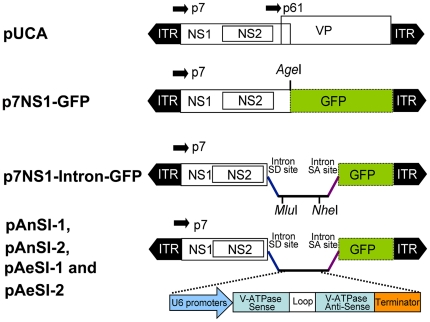
Schematic organization of recombinant AeDNV plasmids. The p7 and p61 viral promoters drive the expression of the NS and VP genes, respectively. In p7NS1-GFP, GFP is fused to the NS1 gene. In p7NS1-Intron-GFP, the chemical intron sequence is inserted in the *Age*I site of the NS1-GFP fusion. RNAi plasmids pAnSI-1, pAnSI-2, pAeSI-1 and pAeSI-2, contain the artificial shRNA expression cassette, which was cloned into the *Mlu*I and *Nhe*I sites of the intron.

The DH5α strain of *Escherichia coli* was used for all cloning procedures and plasmid preparation. Functional features of the plasmids constructed included the following (Figure 1): pUCA is the infectious clone containing the *Ae*DNV genome (3,981 nt) in pUC19; non-structural 1 (NS1) and structural protein (VP) genes were transcribed from the P7 and P61 promoters in the genome, respectively, as described previously [12]. p7NS1-GFP expresses an NS1-green fluorescent protein (GFP) fusion protein from the p7 promoter. The construction of p7NS1-GFP was described in detail elsewhere [11].

*p7NS1-Intron-GFP* was generated by inserting artificial introns into the *AgeI* sites of *pNS1-GFP*. An artificial intron sequence was obtained from the sequence of *pCI-neo* (Promega, Madison, WI, USA) and created by artificial synthesis, and *Mlu*I and *Nhe*I sites were introduced between the 5′-donor site and the branchpoint site to facilitate subsequent insertion of the RNAi expression cassette.

*pAnSI-1, pAnSI-2, pAeSI-1* and *pAeSI-2* were constructed using U6snRNA polymerase III (Pol III) promoters from *Anopheles gambiae* (AnGam-2 Long promoter) and *Aedes aegypti* (Aedes-1 promoter) to express shRNAs as described in detail previously [13]. The shRNA expression cassette was generated by artificial synthesis and was composed of a promoter, the sense *V-ATPase* target sequence, the 7-bp hairpin loop sequence (TCAAGAG) the antisense target sequence, and a poly(T) tract to terminate Pol III transcription. To prepare the shRNA expression vectors pAnSI-2, pAnSI-1 and control vector pAnCSI, the *An. gambiae* Pol III promoter was used to drive siRNA-1, siRNA-2, or scRNA expression and these were subcloned into the *Mlu*I and *Nhe*I sites of *p7NS1-Intron-GFP*. pAeSI-2, pAeSI-1, and control plasmid pAeCSI were constructed using the same approach but using the Pol III promoter of *Ae. aegypti* instead. All of the constructs were confirmed by sequencing (data not shown). The plasmids used in this study are depicted in Figure 1 and sequences of all the shRNA expression cassettes are shown in Text S1.

### Mosquito cell maintenance and transfection

*Ae. albopictus* C6/36 cells (ATCC CRL-1660) were grown at 28°C in Roswell Park Memorial Institute (RPMI) 1640 medium (Gibco BRL, USA) supplemented with 10% heat-inactivated fetal bovine serum (FBS; Gibco BRL, USA). One day before transfection, 2×10^5^ cells per well were plated in six-well plates. The transfection of different RNAi plasmids was performed using Lipofectamine 2,000 (Invitrogen, USA), according to the manufacturer's protocol. Supercoiled plasmids used for transfection were prepared using an OMEGA endo-free Plasmid Purification Kit (Omega, USA). The transfected cells were examined at a wavelength of 490 nm to detect GFP expression.

### Recombinant virus production

Recombinant viruses for RNAi (rAepAnSI-1, rAepAnSI-2, rAepAeSI-1, and rAepAeSI-2) and control (rAepAnCSI and rAepAeCSI) treatments were generated along with the infectious clone pUCA by cotransfecting the corresponding infectious clones, pAnSI-1, pAnSI-2, pAeSI-1, pAeSI-2, pAnCSI, or pAeCSI, with helper plasmid pUCA into C6/36 cells according to the manufacturer's protocol (the cotransfection concentration ratio was 2∶1). After a 5 day incubation, MDV-infected cells were harvested using cell scrapers, lysed by freezing and thawing, and then centrifuged for 10 min at 3,750 rpm. The supernatants were kept as recombinant virus and wild-type AeDNV mixed stocks.

### Mosquito maintenance and transduction

The *Ae. albopictus* strain used in this work was obtained from the Center for Disease Control and Prevention of Guangdong Province. Mosquitoes were maintained at 27°C with 70–80% relative humidity and a 16 h: 8 h photoperiod. Larvae were fed on yeast powder, while adults were maintained on a 10% sugar solution.

To minimize the effect of salt concentration on larval susceptibility to infection [11], [14], 1,000 second-instar *Ae. albopictus* larvae were exposed to recombinant virus rAepAnSI-1, rAepAnSI-2, rAepAeSI-1, or rAepAeSI-2 mixed stocks by introducing them into the beaker that contained 100 ml deionized water and 5 ml of the mixed virus stocks, while rAepAnCSI, or rAepAeCSI mixed stocks were used as negative controls. The blank control group, which received no virus, was exposed to C6/36 cell culture medium in identical conditions to the treatment groups.

After incubation for 24 h at 28°C, the larvae were transferred back to the pans and fed regularly. Once the fluorescent larvae were detected postexposure, they were separated into an individual test plastic cup to facilitate the following continuous observation for detection of portal of entry and tissue tropisms of recombinant virus in *Ae. albopictus* larvae. The same transduction was repeated, but to test of the knockdown of *V-ATPase* in *Ae. albopictus* larvae 3 days post-transduction, the fluorescent larvae were detected and separated into three groups according to the location of GFP expression: in the anal papillae infection group (API), GFP expression was restricted to the anal papillae of the larvae; in the systemic infection group (SI), GFP expression was distributed throughout the body; and in the systemic RNAi test group (SRT), GFP expression was restricted to the anal papillae, which were removed from the body, while the rest of the body was retained for RNAi testing. Each experiment was performed in triplicate. Fluorescent signals of the fusion protein were observed under an inverted fluorescence microscope, and photographs were made using a Nikon ACT-2U digital camera. Data were processed and superimposed using Adobe Photoshop 7.0 software (Adobe Systems Inc., San Jose, CA).

### RNA extraction, reverse transcription (RT) and quantitative real-time PCR

Total RNA was extracted from the different groups of GFP-expressing mosquito larvae and cells at 12 h, 24 h, 48 h, 72 h, and 96 h post transfection using the Total RNA Kit I (Omega, USA). Any residual DNA was removed with RNase-free DNase treatment. First-strand cDNA was synthesized using an oligo (dT) 18 primer and the M-MLV RTase cDNA Synthesis Kit (Takara, Japan). *V-ATPase* mRNA levels were compared with those of *beta-actin* (Accession no. DQ657949). Gene-specific primers were designed using Beacon Designer software 7.5 (Premier Biosoft International, Palo Alto, CA, USA). Primers specific for the *V-ATPase* gene were: V-ATPase-F, 5′-ACGTATCTATGATGGCTGATTCGACCTCTC-3′; and V-ATPase-R, 5′- ACCGACGATGGACACCGAACCTTC-3′, generating a product of 196 bp. Primers used for *beta-actin* were: *β-actin-F*, 5′-CCTGGGTATGGAAGCCTGCGGTATC-3′; and *β-actin-R*, 5′-GGCAATGATCTTGATCTTCATGGTGGATGG-3′, with a product of 195 bp.

Reactions were performed using a RealMasterMix (SYBR Green) (Tiangen, China) and run on MX3005P Real Time PCR System (Stratagene, La Jolla, CA, USA) according to the instructions of the manufacturer. Each reaction contained 10 µl of enzyme mix, 0.15 µM of each primer and 2 µl of DNA solution along with buffer in 20 µl of reaction volume. The PCR program used was: denaturation at 95°C for 1 min, followed by 40 cycles of 95°C for 15 s, 53°C for 30 s, and 68°C for 30 s. Each sample was assessed in triplicate. The real-time PCR results were analyzed using the 2^-ΔΔCT^ method as described [15].

### Western blots

Cells were harvested at the indicated time points after viral infection and total protein was extracted from the cells and mosquito groups using a Total Protein Extraction Kit (Keygen, China) resolved on a 12% sodium dodecyl sulfate (SDS)–polyacrylamide gel and transferred onto a polyvinylidene fluoride (PVDF) membrane. Rabbit anti-V-ATPase polyclonal IgG and mouse monoclonal anti-β-actin antibodies (GeneScript, USA) were used as the primary antibodies (1∶600 dilution) and horseradish peroxidase (HRP)-conjugated goat anti-rabbit and goat anti-mouse IgG (Sigma, USA) were used as the secondary antibodies, respectively.

### Effect of V-ATPase knock down on the lifespan of *Ae. albopictus* larvae

The copy numbers of recombinant virus and AeDNV in the three mixed viral stocks were confirmed by SYBR green-based real-time PCR, as previously described [16]; however, the primer sequences used for recombinant AeDNV were changed (sense, 5′-AGCAGAATCATGGCAGACAG-3′; and antisense, 5′-TACACCGGTAGCGTAGTTGC-3′) and the copy number of recombinant virus and AeDNV were adjusted to the same ratio (recombinant virus copy number: AeDNV copy number  =  1∶5) by adding different volumes of pure AeDNV.

Newly hatched first-instar larvae (1,200 total) were randomly divided into six groups: four recombinant virus experimental groups from I-IV (treated with recombinant mixed stocks rAepAnSI-1/AeDNV, rAepAnSI-2/AeDNV, rAepAeSI-1/AeDNV, or rAepAeSI-2/AeDNV) a wild-type AeDNV-treated group, and a control group. Larvae in all four treatment groups were exposed to the same concentration of mixed recombinant virus or wild type stocks to a total volume of 10 ml, and food was withheld for 24 h. The control group, which received no virus, was exposed to C6/36 cell culture medium in identical conditions to the treatment groups. After 24 h, mosquito larvae were transferred into dechlorinated tap water and fed regularly. Larval mortality was scored every 12 h for 15 days.

The median survival time (LT_50_) was calculated from the time-mortality curve of larvae that were infected by recombinant virus and wt virus at a concentration of 1.0×10^10^ copies/ml.

### Statistical analysis

Survival curves were formulated using the Kaplan-Meier test. The log-rank test was used to analyze the differences between survival curves. LT_50_ values were determined by probit analysis. The LT_50_ values were compared between different treatments by a one-way analysis of variance (ANOVA) followed by the Fisher's least significant difference test (LSD). P-values <0.05 were considered to be statistically significant. The SPSS computer software version 17.0 (SPSS Inc., Chicago, IL) was used for data analysis.

## Results

### Generation of the Pol III-mediated intronic shRNA expression vector

**Figure 2:**
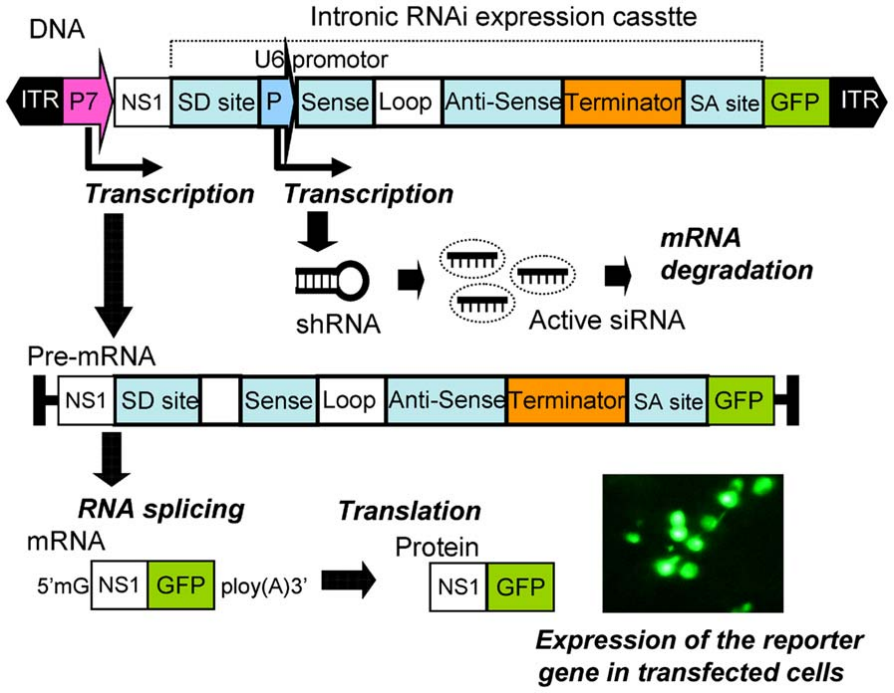
Pol III-mediated intronic siRNA expression vector. Intronic shRNA is transcribed by the U6 promoter accompanied by a pre-mRNA transcribed by the p7 promoter. After pre-mRNA splicing, the exons are ligated to form a mature mRNA. For NS1-GFP fusion protein synthesis, the shRNA is further processed into mature siRNA capable of triggering post-transcriptional gene silencing.

We inserted the Pol III promoter-driven shRNA expression cassettes into a chimeric intron composed of the 5′ donor site from the first intron of the human *beta-globin* gene and the branch and 3′ acceptor site from the intron of an immunoglobulin gene heavy chain variable region [17]. This design takes advantage of the natural process of pre-mRNA splicing, by which introns are removed from pre-mRNA transcripts [18]. NS1 protein is preserved in its entirety in the recombinant genome as the NS1-GFP fusion protein, because of the multiple activities that this densovirus replication initiator protein possesses. Moreover, GFP also provides a robust marker for the recombinant vector-transfected cells *in vitro* and *in vivo*. For the intronic shRNA expression vector, if the artificial intron is not removed in mosquito cells, the frame-shift mutation in the fused GFP marker will preclude fluorescence. We detected GFP in all shRNA expression vector transfected cells, confirming the function of the artificial intron in mosquito cells (Figure 2).

### Knockdown of V-ATPase mRNA and protein expression in C6/36 cells

The direct visualization of green fluorescence allowed us to detect recombinant plasmids that were expressed in mosquito cells. The first fluorescent cells were observed as early as 4 h after transfection, but maximum levels of expression were observed about 48 h later. At 60 h post-transfection, the efficiency was determined by counting GFP-positive cells and total cells from six random fields for each condition. More than 96% transfected cells were GFP-positive determined by Fluorescence inverted microscopy.

**Figure 3:**
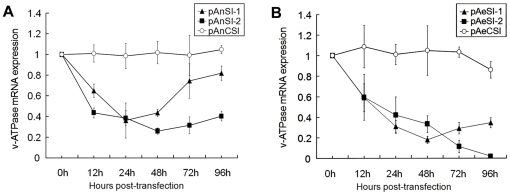
AeDNV-delivered recombinant siRNA inhibits V-ATPase mRNA expression in mosquito cells. Analysis of V-ATPase mRNA expression in *Ae. albopictus* C6/36 cells after transfection with recombinant siRNA vector. (A) *An. gambiae* U6 promoter-driven siRNA vector. (B). *Ae. aegypti* U6 promoter-driven siRNA vector. Error bars represent the standard deviation of the 2^-△△CT^ values for V-ATPase mRNA expression in the C6/36 cell line as evaluated by real-time RT-PCR.

To compare and validate the effect of the different RNAi vectors and determine the time-response effect of silencing V-ATPase, C6/36 cells were transfected with pAnSI-1, pAnSI-2, pAeSI-1, or pAeSI-2, and the expression of V-ATPase was detected by real-time PCR and western blotting at 12, 24, 48, 72, and 96 h post-transfection. Real-time PCR analysis showed that the silencing effects of pAnSI-1, pAnSI-2, pAeSI-1, and pAeSI-2 on C6/36 cells varied. Of these constructs, *An. gambiae* Pol III promoter-driven RNAi vectors pAnSI-1 and pAnSI-2 exhibited significant silencing effects at all time points. The inhibition ratio of pAnSI-1 was 35.03±6.35% (p<0.01) at 12 h post-transfection and reached a peak at 24 h (63.85±16.85%) (p<0.01). The levels of inhibition declined between 48 h (56.48±3.56%; p<0.01) and 96 h (18.21±6.87%; p<0.01), respectively. For pAnSI-2, effective inhibition was observed as early as 12 h (56.51±4.75%; p<0.01) and then increased to 74.06±2.97% (p<0.01) at 48 h. This was followed by a constant decline in mRNA expression at 72 h (68.49±8.07%; p<0.01) and 96 h (59.62±4.62%; p<0.01) after transfection. In contrast, no significant expression changes were observed in the pAnCSI-control group (p > 0.5) (Figure 3A, graph).

However, the *Ae. aegypti* Pol III promoter-driven RNAi vectors pAeSI-1 and pAeSI-2 appeared to be more effective at silencing genes than those driven by the *An.gambiae* Pol III promoter. Analysis of cells transfected with pAeSI-1 indicated that *V-ATPase* knockdown was evident at 12 h (41.02±13.58%; p<0.01) and peaked at 48 h (82.09±3.42%; p<0.01) post-transfection in these cells. Cells transfected with pAeSI-2 exhibited sustained silencing of *V-ATPase* expression. Maximum down-regulation of V-ATPase by pAeSI-2 exceeded 90% at 96 h (98.13±14.1%; p<0.01) post-transfection (Figure 3B, graph).

**Figure 4:**
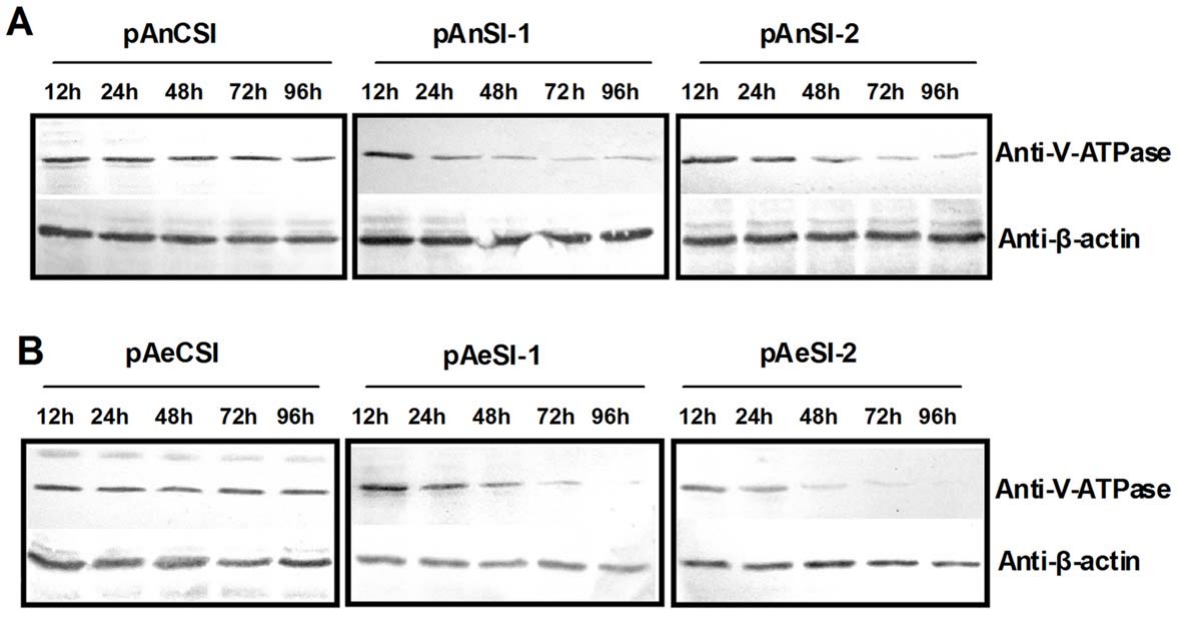
AeDNV-delivered recombinant siRNA inhibits *V-ATPase* expression in mosquito cells. Western blot analysis of *V-ATPase* expression and β-actin (loading control) protein levels in *Ae. albopictus* C6/36 cells after transfection with recombinant siRNA vector. (A) *An. gambiae* U6 promoter-driven siRNA vectors. (B). *Ae. aegypti* U6 promoter-driven siRNA vectors.

Western blot data also indicated that both *An. gambiae* and *Ae. aegypti* Pol III promoter-driven RNAi vectors effectively inhibited *V-ATPase* protein expression in these cells. As expected, *V-ATPase* inhibition increased over time (Figure 4). Notably, pAeSI-2 *V-ATPase* protein expression dropped to undetectable levels at 96 h post-transfection (Figure 4B, graph).

### Portal of entry and tissue tropisms of recombinant virus in *Ae. albopictus* larvae

*Ae. albopictus* larvae were exposed to recombinant virus mixed stocks. GFP marker expression was examined under fluorescence microscopy. The newly emerging GFP-positive larvae were separated continuously from 1 to 3 days post-exposure. The GFP expression was observed in 75.1%, 71.3%, 72.8%, and 78.4% larvae in rAepAnSI-1, rAepAnSI-2, rAepAeSI-1 and rAepAeSI-2 respectively. In the different groups, respectively, 39.3%, 41.9%, 41.3% and 45.1% larvae first showed GFP in the anal papillae; 12.8%, 15.3%, 13.0%, and 16.2% within the bristle cell; 9.8%, 7.9%, 8.5%, and 9.0%, at the base of an anal papilla. Other tissue locations accounted for 6.2%, 6.0%, 7.4%, and 7.1% of the primary infection sites. Furthermore, 28.5%, 24.1%, 26.8%, and 29.3% of larvae showed primary infection in more than one tissue site (Table 1). Investigation into the dissemination of recombinant virus in separated individual mosquitoes was based on daily monitoring of GFP expression. Nearly 83.1%, 87.7%, 92.3%, and 94.7% of larvae developed other infected tissues, including muscle fibers, the midgut, salivary glands, nerves, the malpighian tubule, the foregut and hindgut, and others. Only 1.7%, 0.9%, 2.1%, and 1.5% of larval infection was restricted to the anal papillae, or they lost their infected anal papillae, which delayed or prevented further dissemination.

**Table 1:** Location and frequency of portal of entry in first-instar Ae. albopictus larvae.

Primary infection site	GFP larvae number infected with recombinant virus				
	rAepAnSI-1	rAepAnSI-2	rAepAeSI-1	rAepAeSI-2	Total
Anal papillae (SA*)	393(244)	419(235)	413(217)	451(243)	477(290)
Bristle cell (SA)	328(169)	353(178)	330(189)	362(190)	400(206)
Base of anal papillae (SA)	98(24)	109(27)	125(34)	130(32)	144(31)
Other** (SA)	69(11)	71(9)	48(9)	59(9)	70(20)
Multiple sites	285	241	268	293	279

*SA  =  site alone, the location indicated was unique site of GFP expression observed.

**All locations other than the three types of primary infection indicated in the table.

### Knockdown of V-ATPase in *Ae. albopictus* larvae

**Figure 5:**
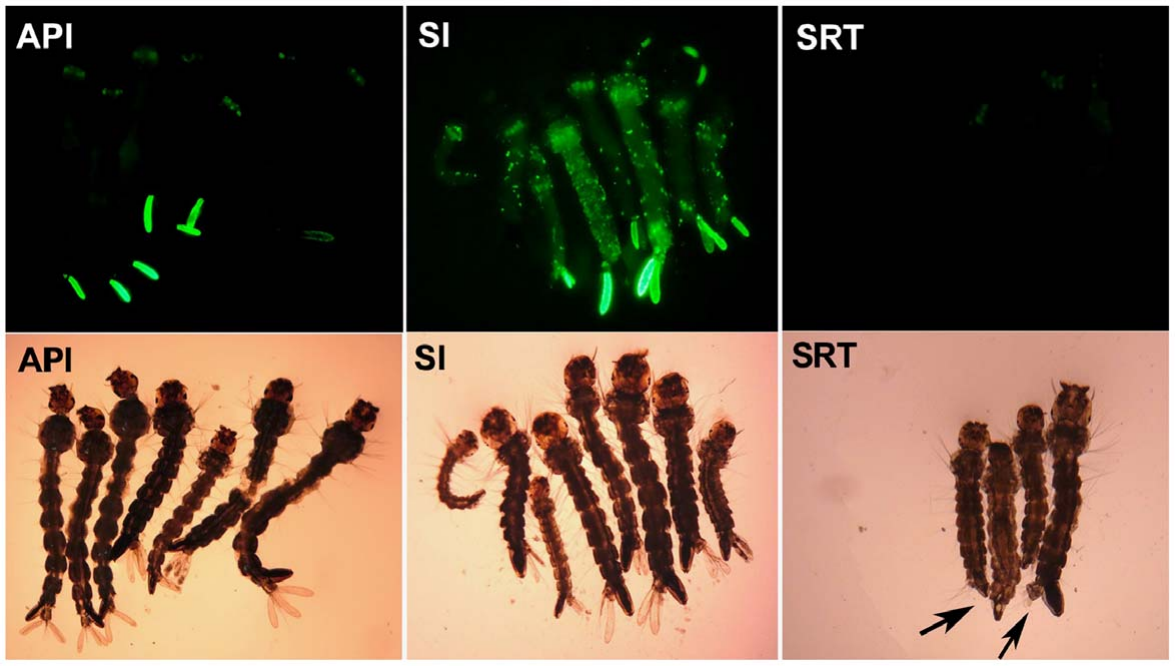
Grouping of mosquito larvae by GFP expression location. In the systemic infection group (SI) GFP expression was distributed throughout the body. In the anal papillae infection group (API) GFP expression was limited to the anal papillae. In the systemic RNAi test group (SRT) The GFP anal papillae were removed (arrow shown).

**Figure 6:**
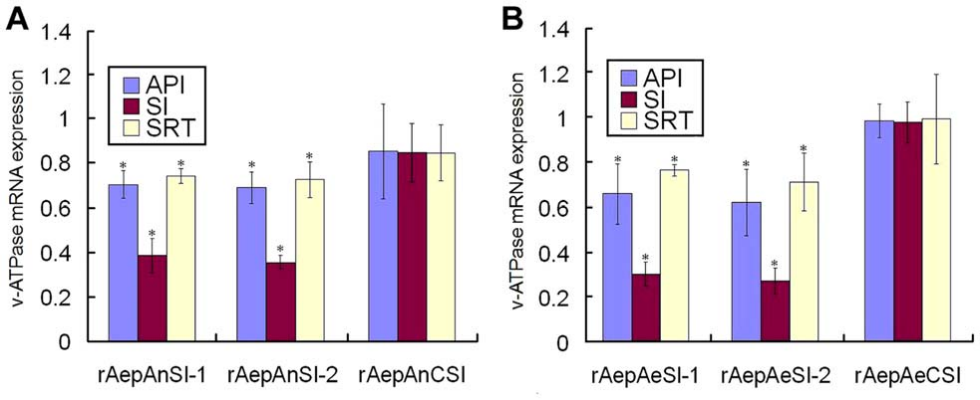
AeDNV-mediated expression of recombinant siRNA inhibits *V-ATPase* mRNA expression in mosquito larvae. Analysis of *V-ATPase* mRNA expression in *Ae. albopictus* larvae in various expression level groups after expression of recombinant siRNA directed against *V-ATPase* driven by: A, *An. gambiae* U6 promoter; or B, *Ae. aegypti* U6 promoter. Error bars represent the confidence intervals of 2^-ΔΔCT±s^ for *V-ATPase* mRNA expression data collected at each time point. s  =  the standard deviation of the ΔΔCT value. *P<0.05.

**Figure 7:**
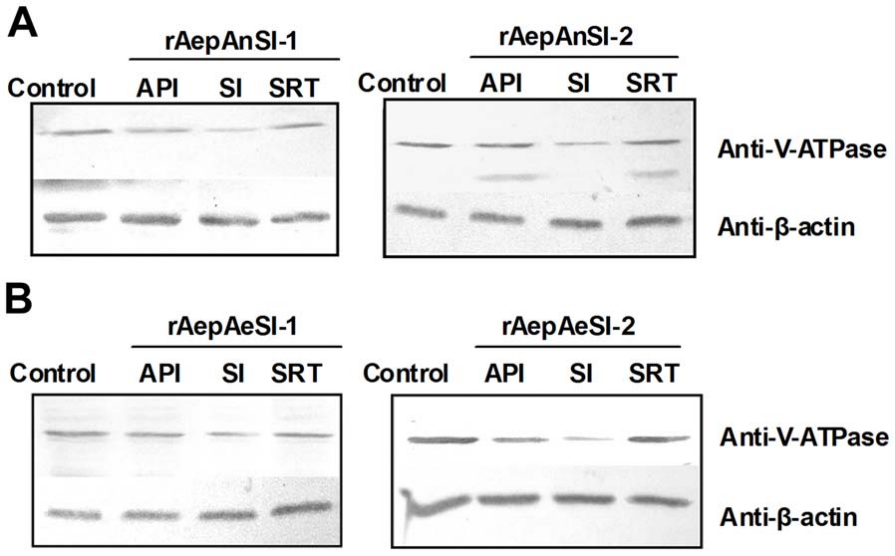
AeDNV-mediated expression of recombinant siRNA inhibits V-ATPase expression in mosquito larvae. Western blot analysis of V-ATPase and β-actin (loading control) protein levels in API, SI, and SRT groups transfected with siRNA driven by the: A, *An. gambiae* U6 promoter or B, *Ae. aegypti* U6 promoter.

The anal papillae of mosquito larvae are classic transport epithelia that can absorb inorganic ions from extremely dilute external media and control the ion balance between the mosquito's hemolymph and its surrounding environment [19], [20]. Anal papillae are the major portal of entry of AeDNV, with dissemination to the whole body occurring from there [16]. However, in some cases the anal papillae present a barrier to virus dissemination, in which case the infection remains restricted to the anal papillae. In these instances, whether the recombinant virus restricted to the anal papillae can also result in a whole body RNAi response is still unknown. To explore this possibility, anal papillae from mosquitoes with infection restricted to the anal papillae were removed and the rest of the body was collected to test the SRT group for whole body RNAi response. Figure 5 shows the different groups being analyzed for this purpose. The effect of recombinant virus on *V-ATPase* knockdown was investigated by real time PCR and western blot. Triplicate experiments showed that the *An. gambiae* Pol III promoter-driven RNAi vectors rAnpAnSI-1 exhibited reductions in *V-ATPase* gene expression of 29.77±6.1% (p<0.01), 61.47±7.51% (p<0.01) and 25.9±3.16% (p<0.05) in the API, SI, and RT groups, respectively, while the same groups infected with rAnpAnSI-2 exhibited reductions of 31.22±7.22% (p<0.01) 64.56±2.97% (p<0.01) and 27.57±7.97% (p<0.05), respectively (Figure 6A). As expected, *Ae. aegypti* Pol III promoter-driven RNAi vectors showed higher rates of *V-ATPase* mRNA inhibition *in vivo* than that of *An. gambiae* Pol III promoter-driven rAepAnSI-1, reducing larval expression by 34.14±13.41% (p<0.01) 69.96±5.30% (p<0.01) and 23.66±2.65% (p<0.05) in the API , SI, and RT group, respectively. rAepAnSI-2 exhibited the strongest inhibitory effect on *V-ATPase* mRNA, as it reduced expression by 38.04±14.70% (p<0.01), 72.87±5.74% (p<0.01) and 28.79±12.81% (p<0.05) in the SI, API, and RT groups, respectively (Figure 6B). These data were confirmed by Western blot analysis (Figure 7, graph), which suggested that the siRNA blocked expression of *V-ATPase* at the protein level in mosquito larvae.

### Knockdown on the lifespan of *Ae. albopictus* larvae

**Figure 8:**
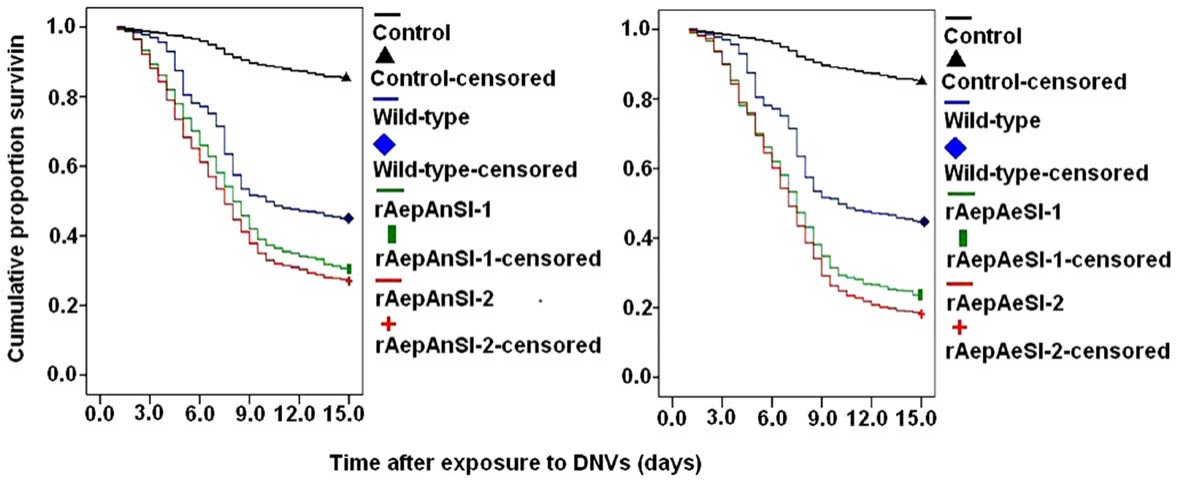
The cumulative proportion of surviving *Ae. albopictus* after first-instar larvae were exposed to doses of wt-type AeDNV and four kinds of recombinant AeDNV mixed stocks at 10^10^ copies/ml.

To study the pathogenicity of recombinant densovirus in *Ae. Albopictus*, newly-hatched first-instar larvae were naturally infected by introduction of the recombinant virus mixture into the water, and their survival was recorded. Figure 8 shows the cumulative proportion of *Ae. Albopictus* that survived for longer than 15 days when exposed to 1×10^10^ copies/ml of densovirus for 48 h. The survival of mosquitoes exposed to rAepAnSI-1, rAepAnSI-2, rAepAeSI-1, or rAepAeSI-2 was significantly different from those exposed to wild-type AeDNV (log rank P<0.05). The LT_50_ values of recombinant AeDNV-exposed mosquitoes were 8.00 (rAepAnSI-1), 7.5 (rAepAnSI-2), 7.5 (rAepAeSI-1) and 7.0 days (rAepAeSI-2) (Table 2). These LT_50_ values were significantly lower than wild-type-exposed mosquitoes (10.0 days). In particular, larvae treated with rAepAeSI-2 mixed stocks exhibited the highest pathogenic effects. Therefore, the increased mortality observed in the recombinant virus-infected larvae was caused by reduced *V-ATPase* expression in larval cells as a result of the RNAi treatment.

**Table 2:** LT50 of virus-treated *Ae. albopictus* larvae.

Group	LT50 (days) (95% CI)
Wild-type	10.00 (7.92–12.08) a
rAepAnSI-1,	8.00 (7.51–8.50) b
rAepAnSI-2	7.50 (7.01–7.99) b
rAepAeSI-1	7.50 (7.10–7.90) b
rAepAeSI-2	7.00 (6.64–7.36) c

The LT50 values were calculated from 200 larvae with each tested virus in triplicate. Means within the same column with different letters are significantly different (p<0.05).

## Discussion

Currently, electroporation, lipid-based transfection reagents, and nanoparticles are commonly used to transfer RNAi molecules into cultured mosquito cells. However, despite growing interest in the application of siRNAs for mosquito gene function analysis or vector control, *in vivo* delivery has been difficult. Direct injection of dsRNA is the most commonly used delivery method for *in vivo* siRNA delivery [21]–[25]. However, this technique is technically demanding and the relatively short half-lives of the delivered dsRNA or siRNA limit their utility [26]. In contrast, viral-based shRNA expression systems have been developed to overcome extracellular and intracellular barriers. In addition, viral-based shRNA delivery has the advantages of easy manipulation, higher transfection efficiency, longer-term expression, and more persistent silencing effects *in vivo* [27]. There are now many successful examples of the use of viral vector-mediated RNAi to inhibit gene expression in animal models of disease [28]–[30]. In addition, this study describes a novel MDV-based siRNA delivery system for mosquito RNA interference applications.

Although their biological characteristics make MDVs attractive vectors for gene transfer in mosquito cells, their relatively small genome has been a major obstacle to application. Insert size testing suggests that 4,100–4,400 bp is the optimal genome size for packaging. With the essential MDV inverted terminal repeats (ITRs) 3′ and 5′ untranslated regions, and NS1 elements, the expression cassette can easily surpass the packaging limits [9]. This poses a problem for large shRNA expression cassettes. As is well known, permanent gene suppression can be achieved by siRNAs as stem-loop precursors transcribed from an RNA Pol II or Pol III promoter-based vector [31], but this size limit excludes most Pol II promoters if the entire NS1 protein is retained for replication of the recombinant virus genome. Another frequently used strategy is inversion of the Pol III promoter-driven shRNA cassette in the 3′ end of the genome [32]. However, in the MDV genome, this region contains the sequences necessary for the termination of viral mRNAs [9]. To solve this, we inserted the Pol III promoter-driven shRNA expression cassettes into a chimeric intron. When this intron was inserted into the protein coding regions of the MDV, mature virus mRNA sequences were unaltered after splicing, regardless of the shRNA expression cassette used. Our results also demonstrated the advantages of preservation of the *NS1* gene and IRS in all recombinant constructs in which NS1 fusion protein and IRS mediate the excision and replication of the recombinant genome in host cell. The continuously increasing copy number of the self-replication vector may contribute to highly efficient and constant down regulation in C6/36 cell than the common RNAi plasmid vector.

Our results demonstrate that the incorporation of an intronic strategy offers a new paradigm to overcome MDV vector size limitations for the efficient use of RNAi in mosquitoes. This result suggests that Drosha processing of the shRNA is relatively efficient even when the shRNA cassette is in an intron. The success of vectors that incorporate a synthetic intron also indicates that the conserved sequences for mRNA splicing (5′ donor, branch, and 3′ acceptor sites) suffice for the efficient processing of pre-mRNAs.

Recently, the U6snRNA promoters of *Ae. aegypti* and *An. gambiae* were characterized for the expression of shRNA targeting firefly luciferase to mediate knockdown of a co-transfected luciferase reporter gene in mosquito cells [13]. Because genome information is lacking for *Ae. albopictus*, and the U6snRNA promoters have not been cloned for this species, the U6snRNA promoters of *Ae. aegypti* and *An. gambiae* were used in this study to express shRNA in *Ae. albopictus* cells, and the gene-silencing effects of these two shRNA expressing cassettes were evaluated and compared.

The characteristic RNAi effects were compared between experiments using the *An. gambiae* U6 promoter and those using the *Ae. aegypti* promoter. Both of the *Ae. aegypti* promoter-based constructs exhibited increased silencing compared with the *An. gambiae*-based constructs, whether used in cultured cells or larvae. In particular, the pAeSI-2 construct exhibited more rapid and sustained silencing of *ATPase* expression in cells. This could be due to the genetic linkage similarity of mosquitoes, as *Ae. aegypti* is closely related to *Ae. albopictus* than to *An. gambiae*. Furthermore, a previous study showed that the *An. gambiae* U6 promoter is more effective at driving RNAi-mediated gene knockdown in AG-55 *An. gambiae* cells [13]. The characteristics of the promoters used in this study should be useful for many mosquito system applications, including functional genomic experiments, and development of RNAi-based strategies for vector control.

*Ae. albopictus* was selected as the target insect because it is an important vector of the Chikungunya (CHIKV) and dengue (DENV) viruses. DENV is considered the most important arbovirus disease on the planet. CHIKV, which has contributed to epidemics in continental Africa and Asia, has caused several serious health and economic problems.

Vacuolar ATPases (V-ATPases) a family of ATP-dependent proton pumps, are commonly found in eukaryotic cell plasma membranes and the membranes of intracellular compartments [33]–[35]. Acidification of intracellular compartments, such as lysosomes, endosomes, and parasitophorous vacuoles, is mediated by V-ATPase, and is essential for entry by many enveloped viruses, as well as invasion into, or escape from, host cells by intracellular parasites [36]. V-ATPases are relatively conserved among mosquitoes, such as *Ae. aegypti*, *Culex quinquefasciatus*, *Ae. albopictus* and *An. gambiae* [37]–[39]. Furthermore, previous reports have shown that V-ATPase has essential functions, making it the best RNAi target for causing lethality in coleopteran insect pests [5]. Therefore, given its essential role in a variety of cellular functions, and its key role in mediating pathogenic invasion, *V-ATPase* is the preferable target gene for pest control and/or disruption of arbovirus transmission.

Systemic RNAi is a phenomenon in which local cellular uptake of dsRNA leads to systemic spreading of the RNAi effect [40], [41]. Insect systemic RNAi has been documented in several insect orders, including Diptera, Coleoptera, Hymenoptera, Orthopetra, Blattodea, Lepidoptera and Hemiptera[42], but there is limited information available on the use of systemic RNAi in mosquitoes.

In our previous studies, we found that the primary portals of AeDNV entry in *Ae. albopictus* were the anal papillae. For most larvae, viral dissemination occurred from infected anal papillae to the whole body. However, in some cases the infection was restricted to the anal papillae, indicating that the anal papillae may be a barrier for recombinant virus dissemination to the whole body [16]. However, our results show that in the SRT groups studied, even if the infected anal papillae were removed, *V-ATPase* was still down regulated in the rest of the body. In other words, shRNA with restricted expression in the anal papillae can lead to an RNAi response in the whole body.

There are still some limitations of the system, e.g. although the pure recombinant virus can be generated by the Sindbis virus expression system, they would lose the ability for secondary transmission that takes place *in vivo* with a defective genome. Therefore, cotransfection with wt virus is necessary for dissemination of recombinant virus *in vivo* from primary infection sites to other parts. However, the problem of the persistence of recombinant virus could be solved effectively by the construction of nondefective hypervirulent strains, and this appears to be feasible with the use of appropriate genetic methods in our ongoing studies.

In conclusion, the ability to reliably deliver RNAi to mosquitoes by recombinant viruses will not only provide a tool for functional analysis of mosquito genes, but will have obvious commercial application as well. RNAi provides a unique mode of action for vector control that could complement current strategies. However, whether this system will become a practical method for insect control remains to be seen. Safety issues will need to be addressed, including possible infection of non-target organisms and the risk of gene flow into non-target organisms [43]. Although MDVs are highly specific for mosquito hosts, and siRNA is highly selective, more data will be needed to support the environmental safety of genetically modified AeDNV. Analogous research regarding the feasibility of using RNAi in the protection of crops against insect herbivores has shown that this strategy holds great promise for the future because it allows for the suppression of a wide range of potential gene targets in insects [7], [44]. Because chemical insecticides have a limited shelf life, and their excessive and repeated use leads to resistance, vector resurgence and environmental problems, effective vector management requires a diversity of tools. We feel that the relative risks of recombinant viruses are far lower than those posed by many chemical insecticides, while offering clear benefits in terms of environmentally safe insect pest control.

## Supporting Information

Text S1 **Sequences of artificial introns and all the shRNA expression cassettes.**
(DOC)
