# Supplementary material for: Development of an Efficient Recombinant Mosquito Densovirus-Mediated RNA Interference System and Its Preliminary Application in Mosquito Control
Source: PLoS One. 2011 Jun 16;6(6):e21329. doi: 10.1371/journal.pone.0021329 (PMC3116905; doi:10.1371/journal.pone.0021329)
Supplement: Text S1 — Sequences of artificial introns and all the shRNA expression cassettes. (DOC) [file pone.0021329.s001.doc]

**Text S1:** Sequences of artificial introns and all the shRNA expression cassettes.

**
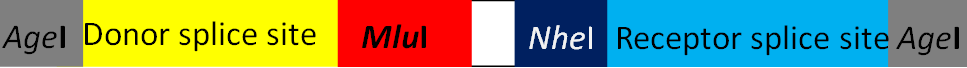
**

**p7NS1-Intron-GFP artificial introns**

**p7NS1-Intron-GFP artificial introns sequences:**

TACGCTACCGGTGTAAGTATCAAGGTTACAAGACAGGTTTAAGGAGACCAATAGAAACTGGGCTTGTCGAGACAGAGAAGACTCTTGCGTTTCACGCGTCCCGGGGCTAGCTGATAGGCACCTATTGGTCTTACTGACATCCACTTTGCCTTTCTCTCCACAGCCGGTCGCCAC


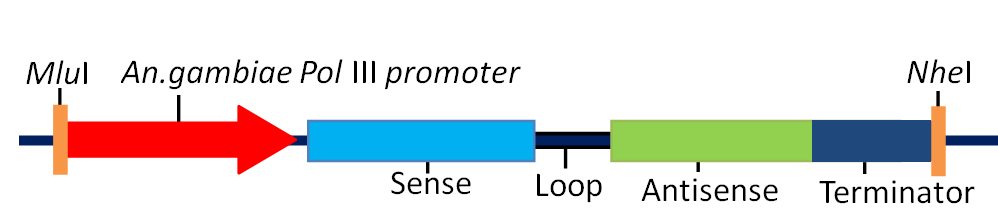


**pAnSI-1 shRNA expression cassettes**

**pAnSI-1 shRNA expression cassettes sequences (208 bp):** TTTC-ACGCGTTTTGTATGCGTGCGCTTGAAGGGTTGATCGGAACCTTACAACAGTTGTAGCTATACGGCTGCGTGTGGCTTCTAACGTTATCCATCGCTAGAAGTGAAACGAATGTGCGTAGGTATATATATGAAATGGAGTTGCTCTCTGCTGTACTAAGGTCAAGGAGATTCAAGAGATCTCCTTGACCTTAGTACTTTTTTTTGCTAGC-TGAT

**
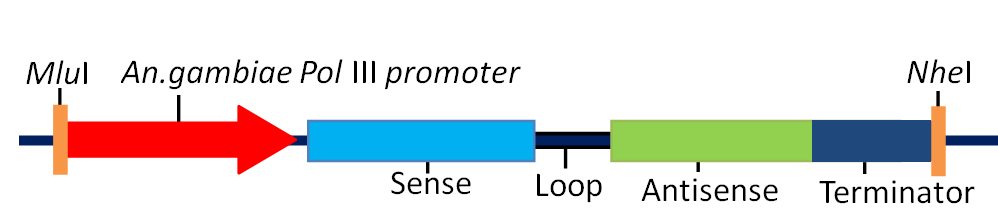
**

**pAnSI-2 shRNA expression cassettes**

**pAnSI-2 shRNA expression cassettes sequences (208 bp):**

TTTC-ACGCGTTTTGTATGCGTGCGCTTGAAGGGTTGATCGGAACCTTACAACAGTTGTAGCTATACGGCTGCGTGTGGCTTCTAACGTTATCCATCGCTAGAAGTGAAACGAATGTGCGTAGGTATATATATGAAATGGAGTTGCTCTCTGCTGCTGAAGGATATTAACGAATCAAGAGTTCGTTAATATCCTTCAGCTTTTTTTTGCTAGC-TGAT

**
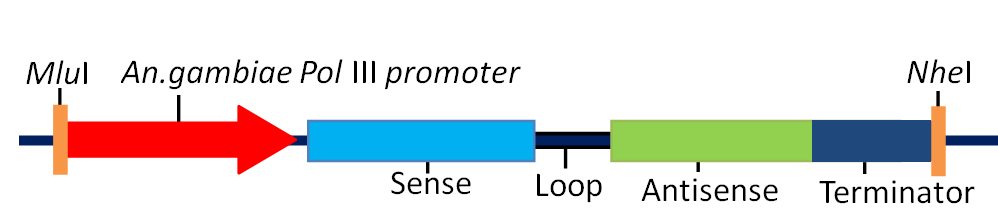
**

**pAnCSI shRNA expression cassettes**

**pAnCSI shRNA expression cassettes sequences (208 bp):**

TTTC-ACGCGTTTTGTATGCGTGCGCTTGAAGGGTTGATCGGAACCTTACAACAGTTGTAGCTATACGGCTGCGTGTGGCTTCTAACGTTATCCATCGCTAGAAGTGAAACGAATGTGCGTAGGTATATATATGAAATGGAGTTGCTCTCTGCTCGACGACTATCGTGCAATTTCAAGAG AATTGCACGATAGTCGTCGTTTTTTTTGCTAGC-TGAT

**
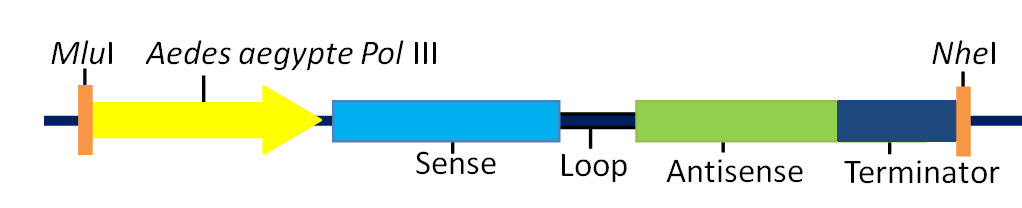
**

**pAeSI-1 shRNA expression cassettes**

**pAeSI-1 shRNA expression cassettes sequences (160 bp):**

TTTC-ACGCGTGAATGAAATCGCCCATCGAGTTGATACGTCCATCCATCGCTAGAACCGCGTTCGCTGTAGAAGACTATATAAGAGCAGAGGCAAGAGTAGTGAAAGTACTAAGGTCAAGGAGATTCAAGAGATCTCCTTGACCTTAGTACTTTTTTTTGCTAGC-TGAT


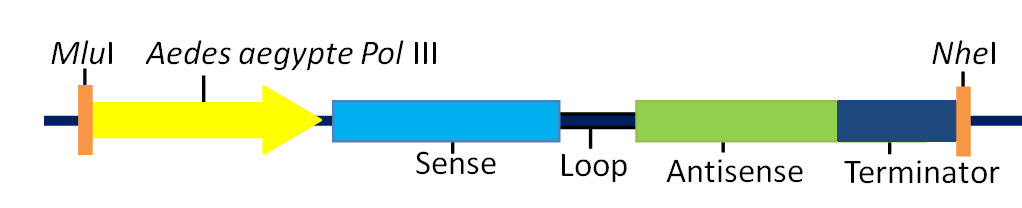


**pAeSI-2 shRNA expression cassettes**

**pAeSI-2 shRNA expression cassettes sequences (160 bp):** TTTC-ACGCGTGAATGAAATCGCCCATCGAGTTGATACGTCCATCCATCGCTAGAACCGCGTTCGCTGTAGAAGACTATATAAGAGCAGAGGCAAGAGTAGTGAAAGCTGAAGGATATTAACGAATCAAGAGTTCGTTAATATCCTTCAGCTTTTTTTTGCTAGC-TGAT


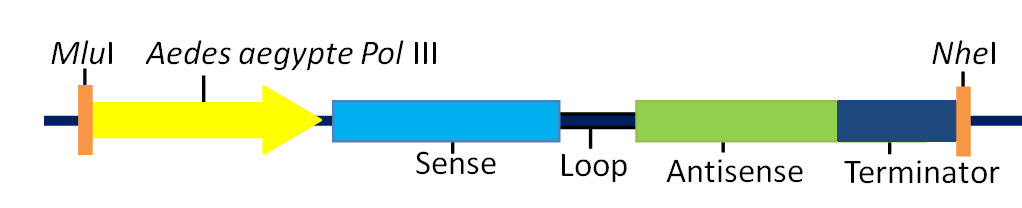


**pAeCSI shRNA expression cassettes**

**pAeCSI shRNA expression cassettes sequences (160 bp):** TTTC-ACGCGTGAATGAAATCGCCCATCGAGTTGATACGTCCATCCATCGCTAGAACCGCGTTCGCTGTAGAAGACTATATAAGAGCAGAGGCAAGAGTAGTGAAACGACGACTATCGTGCAATTTCAAGAGAATTGCACGATAGTCGTCGTTTTTTTTGCTAGC-TGAT

The colors in the figure correspond to the sections colored in the sequences.
